# TFNR2 in Ischemia-Reperfusion Injury, Rejection, and Tolerance in Transplantation

**DOI:** 10.3389/fimmu.2022.903913

**Published:** 2022-07-07

**Authors:** Araz Kouyoumdjian, Jean Tchervenkov, Steven Paraskevas

**Affiliations:** ^1^ Division of Experimental Surgery, Faculty of Medicine and Health Sciences, McGill University, Montreal, QC, Canada; ^2^ Division of General Surgery, Department of Surgery, McGill University, Montreal, QC, Canada

**Keywords:** TNFR2, immune regulation, transplantation, rejection, ischemia reperfusion injury (IRI)

## Abstract

Tumor necrosis factor receptor 2 (TNFR2) has been shown to play a crucial role in CD4+ T regulatory cells (CD4+Tregs) expansion and suppressive function. Increasing evidence has also demonstrated its role in a variety of immune regulatory cell subtypes such as CD8+ T regulatory cells (CD8+ Tregs), B regulatory cells (Bregs), and myeloid-derived suppressor cells (MDSCs). In solid organ transplantation, regulatory immune cells have been associated with decreased ischemia-reperfusion injury (IRI), improved graft survival, and improved overall outcomes. However, despite TNFR2 being studied in the context of autoimmune diseases, cancer, and hematopoietic stem cell transplantation, there remains paucity of data in the context of solid organ transplantation and islet cell transplantation. Interestingly, TNFR2 signaling has found a clinical application in islet transplantation which could guide its wider use. This article reviews the current literature on TNFR2 expression in immune modulatory cells as well as IRI, cell, and solid organ transplantation. Our results highlighted the positive impact of TNFR2 signaling especially in kidney and islet transplantation. However, further investigation of TNFR2 in all types of solid organ transplantation are required as well as dedicated studies on its therapeutic use during induction therapy or treatment of rejection.

## Introduction

Tumor necrosis factor (TNF) signaling is central to many aspects of ischemia-reperfusion injury (IRI) in solid organ transplantation and has a central role in regulating acute and chronic anti-donor immune responses which can determine or limit the functional life of the transplanted organ. TNFα is mainly produced by macrophages and neutrophils during innate immune activation after recognition of either pathogen-associated molecular patterns (PAMPs) or danger-associated molecular patterns (DAMPs) *via* pattern recognition receptors (1). It can also be produced by CD4+ effector T cells and natural killer cells during certain conditions (2). It exerts its actions through binding with its receptors TNF receptor type 1 (TNFR1) and TNF receptor type 2 (TNFR2) as either a transmembrane protein (mTNF) or soluble protein (sTNF) (3, 4). With pertinence to the organ transplant context, TNFR2 is expressed in lymphocytes as well as endothelial cells (5), the latter being the primary donor-recipient interface from the immunological perspective. On the other hand, TNFR1 is ubiquitously expressed on most cell types (6).

The inflammatory phenotypes instigated by TNFα have been studied extensively in the context of innate and adaptive immune cell activation (7–13). Indeed, TNFα signaling through both TNFR1 and TNFR2 has been found to have a central role in many pathological conditions such as autoimmunity, allergy, and malignancy (14–16). Despite TNFα’s key role in mediating and perpetuating pro-inflammatory signals during all stages of the immune activation, increasing evidence has highlighted a strong counterbalancing and immune regulatory role for TNFR2 when compared to TNFR1.

While TNFR2 has been studied extensively in the context of cancer and autoimmunity, there remains paucity of data in the transplant field. The first and most significant immunologic event in the transplant process is ischemia reperfusion injury (IRI). During the ‘reperfusion’ phase of IRI, products of cell death are released into the circulation, stimulating the innate and adaptive immune responses (17). In view of the dichotomous role of TNFα signaling, it is important to better illustrate the role that TNFα-TNFR2 holds in all aspects of solid organ transplantation, beginning with IRI and extending to chronic alloimmune processes throughout the life of the organ.

In this review article, we will aim to summarize the evidence regarding TNFα-TNFR2 signaling in the context of regulatory immune cells as well as summarize the role of TNFα-TNFR2 signaling during IRI, solid organ transplantation, and rejection.

## TNFα Signaling Pathway

TNFα is a type II transmembrane protein which has a characteristic homotrimeric configuration and exists in two forms, membrane-bound and soluble (4, 18, 19). mTNF can be cleaved in its stalk region by the matrix metalloproteinase TNF-alpha-converting enzyme to form sTNF (20). After its release, sTNF can circulate in the body and thus can exert its effects away from its initial site of production. mTNF and sTNF have been involved in differential pathway signaling. mTNF and sTNF both bind to TNFR1, however, mTNF preferentially binds TNFR2 (21). This suggests that activation of TNFR2 associated pathways are mostly stimulated by neighboring cells in a paracrine fashion while activation of TNFR1 *via* sTNF functions in an endocrine manner.

Upon binding of TNFα to TNFR1, signaling *via* the TNFR1-associated death domain is initiated which can in turn activate one of two distinct cellular responses (22). Through its death domain, TNFR1 signaling leads to activation of the downstream canonical NF-κB, mitogen-activated protein kinase (MAPK), and c-Jun N-terminal kinase (JNK) signaling pathways (23). These three signaling pathways promote inflammation and proliferation. On the other hand, TNFR1 can also induce cell death through activation of the caspase cascade (24).

Upon binding of TNFα to TNFR2, the classical and alternative NF-κB signaling pathway will be activated (25). TNFR2 has also been shown to recruit the TNF receptor associated factor 2 (TRAF2) and the single cellular inhibitor of apoptosis 1 or 2 molecules (cIAP1/2) complex more efficiently than TNFR1 (25, 26). This preferential binding to TNFR2 leads to TRAF2-cIAP1/2 complex depletion and subsequent inhibition of downstream TNFR1 inflammatory signaling (26). On the other hand, as TNFR2 lacks a death domain, it has been shown to recruit and activate the AKT pathway which promotes cell survival, migration, proliferation, CD4+Treg function, and an overall cytoprotective phenotype (27).

## TNFR2 in Immune Regulatory Cells

### TNFR2 in CD4+ T Regulatory Cells

**Figure 1:**
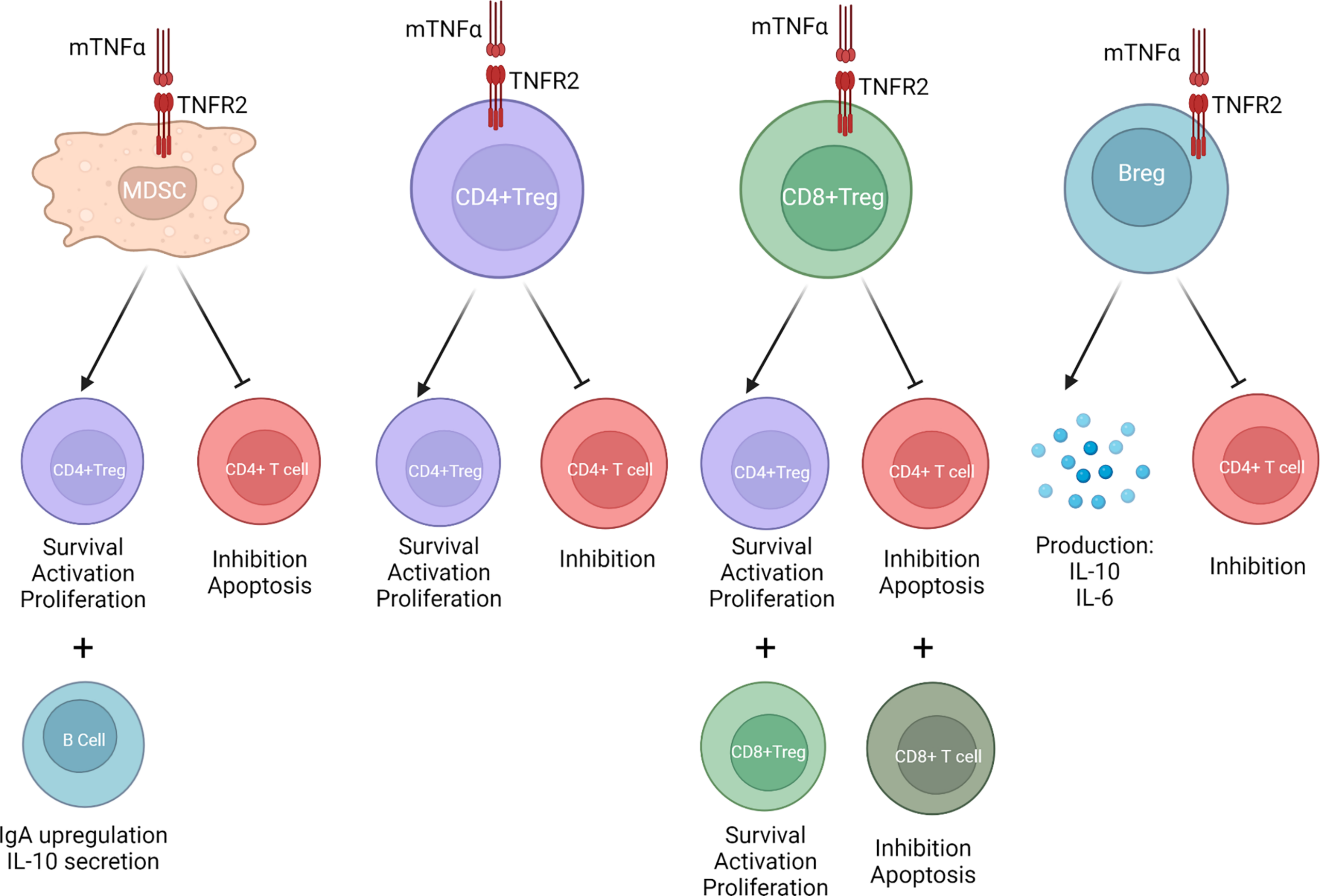
When mTNFα binds to TNFR2, MDSCs, CD4+Tregs, CD8+Tregs, and Bregs are activated. This increases CD4+Treg and CD8+Treg stability and expansion. It stimulates production of anti-inflammatory cytokines such as IL-10 and TGF-β. It inhibits CD4+ T effector cell function. It promotes upregulation of immunoglobulin A (IgA) B cells.

The dichotomous role of TNFα in pro-inflammatory versus regulatory pathways has largely been studied in the context of CD4+ T cells. While initial exposure to TNFα will prompt CD4+ effector functions, prolonged exposure will trigger habituation, downregulation, and exhaustion of CD4+ effector T cell signaling (28). CD4+ T regulatory cells (CD4+Tregs), classically defined as CD4+CD25+CD127- and forehead box P3+ (FoxP3) cells, are a subset of CD4+ T cells which play a crucial role in immune suppression, homeostasis, and self-tolerance through inhibition of CD4+ T effector cell proliferation as well as production of inhibitory cytokines (29, 30). Their dysregulation has been associated with a variety of autoimmune diseases, cancers, as well as solid organ rejection (31–34). Notably, the expression of TNFR2 has been shown to preferentially associate with FoxP3+CD4+Tregs as well as identify a highly suppressive subset of CD4+Tregs in mice as well as humans (35–37). This was shown in experimental models of autoimmune encephalomyelitis, TNFR2 signaling on Tregs has been shown to maintain Treg suppressive activity and protect against disease progression (38). Another functional role which has been found has been CD4+Treg population expansion and proliferation *via* TNFR2 co-stimulation (39). These *in vitro* findings have also been observed *in vivo* murine models of graft-versus-host disease (GvHD). Upon treatment with a TNFR2 agonist, CD4+Treg population expansion and activation was observed (40). Other novel TNFR2 agonists are also being studied as potential therapeutic targets for treatment of autoimmunity and other inflammatory disorders and have been shown to expand highly suppressive CD4+Tregs capable of CD8+ T cell repression *in vitro* (41). CD4+Tregs have also been found to respond to proinflammatory cytokines. Indeed, IL-6 and TNFα have been observed to drive human CD4+Tregs proliferation by increasing TNFR2 expression (42). Recent investigations have shown that binding to TNFR2 on conventional CD4+ T cells increases their interleukin (IL)-2 production which in turn leads to signal transducer and activator of transcription (STAT) 5 phosphorylation and proliferation of neighboring CD4+Tregs (43–45). These findings were confirmed in studies where blocking TNFR2 signaling was shown to increase T helper 17 cell differentiation *via* STAT3 activity and retinoid acid-receptor-related orphan receptor-γt induction (22, 46). TNFR2 has also been studied in the context of thymic CD4+Tregs, or tTregs (formerly called natural Tregs versus peripheral CD4+Tregs, or pTregs (formerly called induced Tregs. In the past, it was believed that TNFR2 signaling was required for optimal tTregs suppressive function under inflammatory conditions alone (47). However, more recent data has put this into question and has rather observed a role for TNFα-TNFR2 signaling in the differentiation and function of pTregs in autoimmunity models (48). On the other hand, there have also been reports that anti-TNFα therapy such as infliximab increase pTreg frequency in human models (49). Together, these data suggest an important role for TNFR2 in CD4+Treg function, however, the exact mechanisms remain elusive, and the discrepancies could be explained by different functionality of pTregs and tTregs in various pathological conditions (**Figure 1**).

### TNFR2 in CD8+ T Regulatory Cells

CD8+FoxP3+ T regulatory cells (CD8+Tregs) are another important class of adaptive regulatory immune cell (50, 51). They are often characterized as CD8+CD25+ Tregs and have similar functions as CD4+CD25+ Tregs (52). They have also been shown to induce tolerogenic antigen-presenting cells as well as induce T effector cell killing in addition to T effector cell function suppression (53). In the context of solid organ transplantation, their capacity to induce tolerance and their role as a potential therapeutic avenue is being increasingly studied (53, 54). In addition, TNFR2 has been found to be a more reliable surface marker than CD25 for characterization of functional CD8+Tregs (51). The induction of CD8+Foxp3+TNFR2+ T cells has also been observed after treatment with anti-CD3 monoclonal antibody treatment in patients with Type 1 diabetes mellitus patients (55). In this study, combined expression of CD8+ with CD25+ identified a highly suppressive CD8+Treg subset which were able to suppress CD4+T cell activity the most. Research has also been performed in the context of hematopoietic stem cell transplantation. In these *in vivo* mice studies, it was suggested that TNFR2+CD8+Tregs can preferentially target allogeneic T cells and thus effectively treat GvHD and rejection (56, 57). However, as in CD4+ T cells, TNFα-TNFR2 expression and signaling within the CD8+ population is varied. TNFR2 is expressed on CD8+ T effector cells and has been shown to be a marker of their proliferative and cytotoxic effects during early phases of the immune response as well as a signal for CD8+ T effector cell apoptosis and activity termination during the later phases of the immune response (51). These diverse functions highlight the complexity required to maintain homeostasis as well as the care that must be taken when studying these cells and developing targeted therapies.

### TNFR2 in B Regulatory Cells

B regulatory cells (Bregs) are a highly diverse suppressive subset of B cells which can arise at any stage in B cell development and have strong suppressive ability, particularly in response to inflammation (58, 59). Bregs have a hallmark production of cytokines such as IL-10. IL-10 release by B cells has been shown to reduce T cell proliferation (60). Bregs have also shown to take part in tolerance induction in solid organ transplantation (61–63). Previously, no common surface marker for Bregs were known. However, TNFR2 expression on B cells has been recently shown to characterize Bregs (64). In this same study, administration of TNFR2 agonist to cell cultures enhanced both production of IL-10 and IL-6 by these B cells. TNFR2 expression has also been related to human memory B cells with suppressive function and increasing evidence suggests that expression of TNFR2 on B cells is characteristic of a suppressive B cell subset (59).

### TNFR2 in Myeloid-Derived Suppressor Cells

Myeloid-derived suppressor cells (MDSCs) are important negative immune regulators and are characterized by a heterogenous population of both immature and progenitor myeloid cells (65). In the context of lung transplantation, granulocyte derived MDSCs have been shown to be correlated with a stable patient phenotype while lower levels have been associated with post-transplant complications (66). The expression of TNFR2 on MDSCs has also been revealed (**Figure 1**). Monocyte-derived MDSCs (Mo-MDSC), which are a subset of MDSC, have been shown to be highly effective in suppressing proliferation of CD4+ T effector cells as well as expanding CD4+Tregs. TNFR2 expression on these Mo-MDSCs has been associated with their generation and function (67, 68). In addition, mTNF has been shown to promote MDSC suppressive function and this activity appears to be mediated *via* TNFR2 expression (68, 69). In cancer mice models, TNFR2 expression on MDSCs has been found to crosstalk with B cells in germinal centers. Interestingly, this interaction has been shown to mediate immunosuppression by upregulating immunoglobulin A (IgA) responses and promoting IL-10 production (70). TNFR2 expression on MDSCs has also been studied in the context of tuberculosis and has been associated as a strong driver of anti-inflammatory function (71).

## TNFR2 Expression and Ischemia Reperfusion Injury

Ischemia-reperfusion injury (IRI) is an inevitable component of organ transplantation and strongly contributes to short and long-term graft outcomes. Increasing evidence now links IRI with activation of the innate and adaptive immune systems (72). During the ischemia phase, adenosine triphosphate (ATP) and glycogen depletion occur, which then leads to mitochondrial dysfunction and ultimately cell death of both endothelial cells and tubular epithelial cells. During reperfusion, there is release of reactive oxygen species, chemokines, cytokines, as well as the products of cell death into the circulation (17). This ultimately stimulates the innate immune response which then enhances activation of the adaptive immune responses and promotes graft allorecognition.

TNFα has been found to be a key cytokine in inducing apoptosis during progression of IRI in many contexts including acute kidney injury (AKI) models (5, 73, 74). In these studies, rat experimental models of AKI were studied and anti-TNFα therapy was found to improve renal IRI recovery. However, these studies lack information on the differential contributions that TNFR1 and TNFR2 hold during IRI.

The differential role of TNFα signaling *via* TNFR1 and TNFR2 has been studied in IRI in the context of arteriogenesis and angiogenesis (75, 76). In these studies, TNFR2 expression on vascular endothelial cells was found to induce endothelial cell angiogenesis, proliferation, survival, and migration while TNFR1 caused endothelial cell apoptosis and inhibition of migration.

Other *in vivo* models of the role of TNFR2 in IRI have also been studied. In mouse hind-limb ischemia models, TNFR2 deletion has been shown to be associated with an increase in inflammatory response as well as in a decrease in post hind limb ischemia recovery (77). This same group has shown that TNFR2 is required in ischemia-induced neovascularization and is protective in human adult myocardial infarctions (78, 79). There have also been studies of IRI in cardiomyocyte-induced ischemia (80).. In these settings, TNFR2 expression on cardiomyocytes was associated with cardioprotection while TNFR1 expression on cardiomyocytes was associated with cardiac dysfunction, fibrosis, and cell death.

Etanercept is a fusion protein consisting of TNFR2 combined with the immunoglobulin G1 fragment crystallizable region, developed for use in autoimmune disorders (81). It is the first therapeutic agent to specifically target TNFR2 signaling, by simulating TNFR2 shedding. While data is limited in the transplant context, etanercept use has been shown to be advantageous in limiting sequelae of IRI in various contexts, including kidney (82), heart (83, 84), brain (85, 86), and intestine (87, 88).

## Therapeutic Use in Islet Transplantation

**Figure 2:**
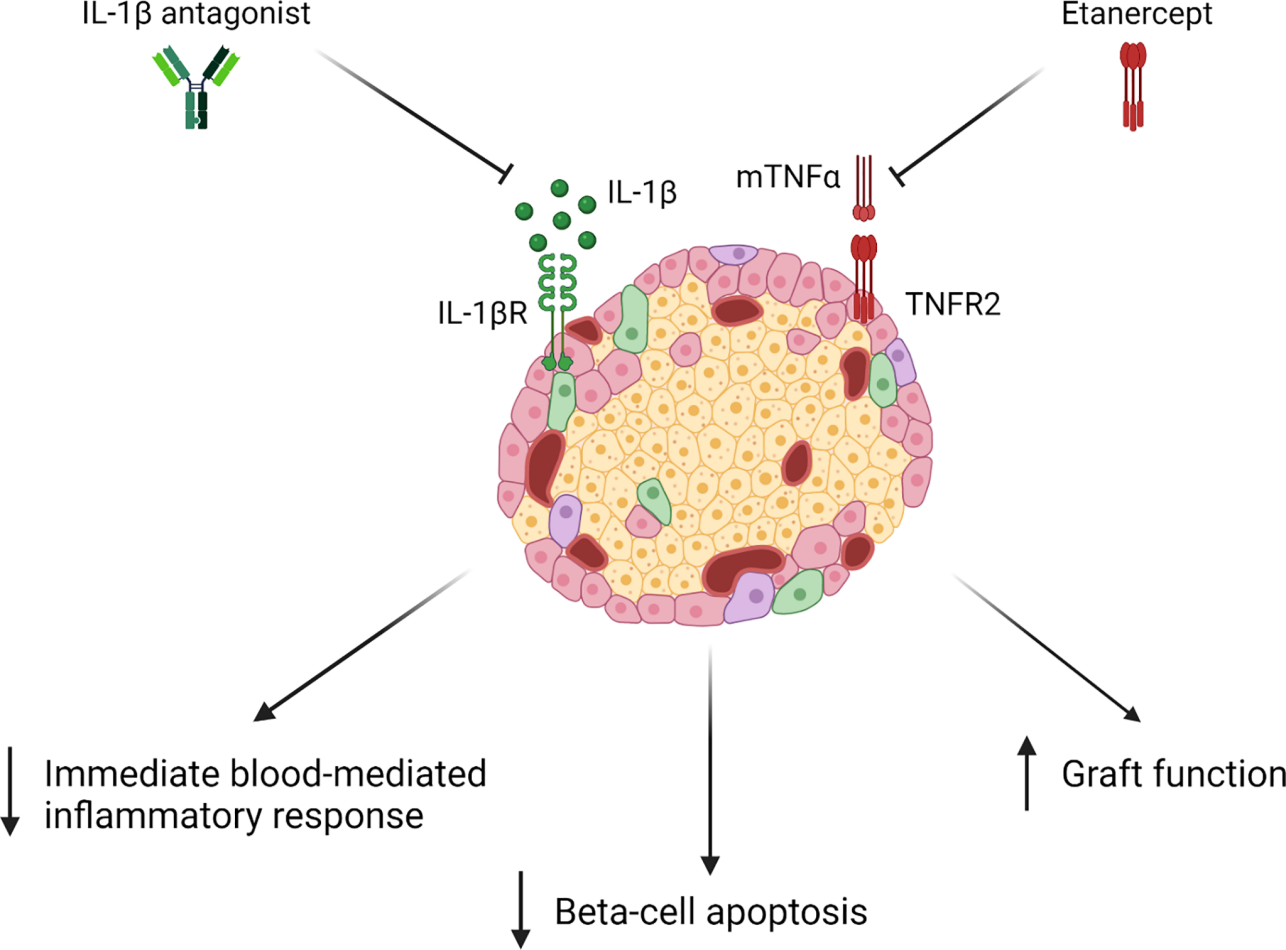
Upon administration of Etanercept with an IL-1β antagonist, studies have shown a decrease in immediate blood-mediated inflammatory response (IBMIR), beta-cell apoptosis, and an improvement in graft function (84–86).

Islet transplantation is a treatment of choice in many jurisdictions, particularly for diabetes patients with severe hypoglycemic unawareness, thanks to improvements in insulin independence rates among recipients. Infusion-related cytokine release and neutrophil chemotaxis has long been appreciated as a cause of islet graft loss, leading to a syndrome known as the immediate, blood-mediated inflammatory response (IBMIR) (89). Furthermore, studies of islet cell apoptosis following isolation suggested that TNFα production by the cell preparation was associated with higher levels of cell death (90). In experimental models, etanercept use was associated with improved function and lower rates of beta-cell apoptosis, particularly when used in conjunction with an anti-IL-1β agent (91). Early adoption in the clinical setting suggested that etanercept use was associated with improved graft function and a higher degree of success following a single infusion of donor islets (92, 93). This was confirmed in larger analyses as greater experience was gained with multiple immunosuppressive protocols (94), with administration of etanercept being associated with preservation of insulin independence at 5 years post-transplant, compared to protocols using similar induction therapy, but without etanercept (**Figure 2**). More robust evidence of the utility of this approach was seen in multivariable statistical analysis of international registry data, in which etanercept use was associated with a significant reduction in risk of graft failure (95). Similar conclusions were drawn in a systematic analysis of reported clinical data (96). These results cemented etanercept use as a mainstay in peri-transplant immunotherapy in this context (97). Early clinical experience with combination therapy targeting TNFα and IL-1β signaling has also been shown to be safe (98). As newer, stem-cell derived products become available to treat type 1 diabetes mellitus, their use may still be complicated by IBMIR and the effects of IRI on the graft during pre-transplant culture and infusion. While etanercept use may provide an advantage during this period, some evidence suggests a narrow therapeutic window beyond which toxicity may be seen (99).

## TNFR2 in Solid Organ Transplantation and Acute Rejection

Early graft function is crucial to early patient survival and to long term outcomes in many organ transplant contexts, and is closely related to degree of IRI. In kidney transplantation, IRI manifests as delayed graft function (DGF) (100). DGF, which is defined as the need for dialysis after kidney transplantation, affects between 25 and 40% of grafts from deceased donors, and has long been associated with a higher lifetime risk of acute rejection episodes and poorer long term allograft survival (101). In the context of liver transplantation, IRI is linked to increased liver enzymes, higher rates of biliary strictures, increase risk of acute and chronic rejection, and ultimately poorer long-term graft outcomes (102–104). In the context of pancreas transplantation, IRI is associated with pancreatitis, graft thrombosis, as well as graft loss (105). For lung transplantation, IRI increases chance of alveolar lung damage, lung edema, hypoxemia, and graft failure (106). Finally, for heart transplantation, IRI is associated with early graft dysfunction, primary graft dysfunction, and cardiac allograft vasculopathy (107).

As described earlier, TNFα-TNFR2 interactions have been shown to dampen IRI response in a variety of non-transplant contexts. While the role of TNFR2 expression in renal IRI after transplantation hasn’t yet been studied, its role as a marker of graft function has been increasingly explored. *De novo* TNFR2 expression on human tubular epithelial cells was first observed during renal transplant biopsies of acutely rejecting allografts (108). It was also discovered that amount of TNFR2 expression correlated with severity of rejection episode. These findings were confirmed in both human and rat studies where biopsies of renal allografts with acute rejection were taken and showed significantly higher staining for TNFR2 observed on podocytes and renal tubular epithelial cells (109). However, despite TNFR2 expression on tubular epithelial cells correlating with acute rejection episode and severity, no long-term data was available for human patients in studying if TNFR2 was also related to faster resolution or improved return to baseline creatinine. Also, no mechanistic studies were performed during these models and thus these studies could not differentiate if this increase in TNFR2 expression was an instigator of rejection or a response to rejection for its resolution. Finally, TNFR2 expression in peripheral blood sample of the patients was not studied and could have been an interesting addition to assess if any changes in TNFR2 frequency on immune regulatory versus pro-inflammatory cells was observed. An increase in serum TNFR2 expression in patients with acute rejection post kidney transplant has also been observed (110, 111). However, these studies were lacking correlation with long-term graft and patient outcomes as well as was characterization of the cell type which had an increase TNFR2 frequency. Together, these studies suggest a role for TNFR2 measurement in conjunction with serum creatinine as a diagnostic marker of acute rejection however, they lack the mechanistic link between TNFR2 and acute rejection and lack long-term graft and patient outcomes data to further clarify this link.

The mechanistic links and signaling pathways involved in TNFR2 expressing tubular epithelial cells in the context of acute rejection have since been studied (112, 113). TNFR1 signaling was shown to colocalize with apoptosis signal-regulating kinase-1 (ASK1) on glomerular cells and peritubular capillary endothelial cells while TNFR2 colocalized with endothelial/epithelial tyrosine kinase (EKT) on tubular epithelial cells and glomerular cells. While TNFR1-ASK1 were shown to induce proapoptotic pathways, TNFR2-EKT signaling was shown to induce tubular epithelial cell adhesion, migration, proliferation, and survival during acute rejection episodes. The same group revealed an associated upregulation as well as colocalization of TNFα converting enzyme (TACE) with TNFR2 on tubular epithelial cell during acute rejection episode. This suggests a role for TACE to promote TNFR2 shedding and limit proinflammatory TNFα effects during acute rejection episodes (114). More recently, the study (115) reported that TNFR2 signaling induces regeneration of tubular epithelial cells during acute cell-mediated kidney rejection. In this study, a mechanistic link between TNFR2 signaling and induction of tubular epithelial stem cell properties during acute rejection was observed.

While these studies observed an association between TNFR2 expression on tubular epithelial cells and acute rejection, there is paucity of data looking at TNFR2 expression on immune cells during solid organ transplantation. Our group has previously studied the role of the frequency of recipient pretransplant TNFR2+CD4+Tregs as a predictor of post kidney transplant DGF and short to medium-term outcomes (116). Increase pretransplant circulating peripheral CD4+Treg TNFR2 frequency was associated with decreased post-transplant DGF rates and was hypothesized to be linked to its role in inducing and maintaining higher CD4+Treg suppressive function. Also, TNFR2 expression on Mo-MDSCs has been associated with increased CD4+Tregs frequency in human renal transplantation (117).

TNFR2 expression has also been briefly explored in heart transplantation. In cardiac transplant mice models an increase in TNFR2 expression was observed on cardiomyocytes and was associated with an increase in cell cycle entry and proliferation of these cardiomyocytes during acute rejection episodes (118). On the other hand, both TNFR1 and TNFR2 have been associated with graft arterial disease in cardiac allografts (119). Also, in rat cardiac allograft models, treatment with TNFR2 recombinant protein decreased TNFα expression as well as decreased cardiac remodeling (120).

**Figure 3:**
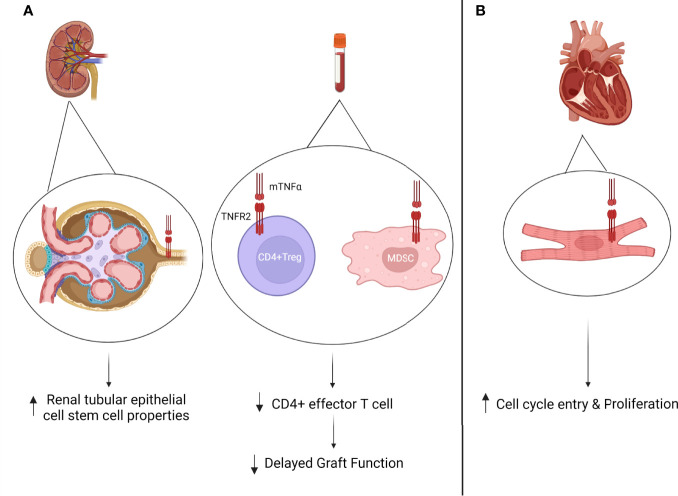
**(A)** TNFR2 expression in kidney transplantation. Increased TNFR2 expression on tubular epithelial cells during acute rejection episode has been associated with renal tubular regeneration (108). Circulating pre-transplant TNFR2 expression on CD4+Tregs has been associated with decreased delayed graft function (DGF) rates (109). Circulating post-transplant MDSCs has been associated with increased CD4+Treg frequency (105). **(B)** Increased TNFR2 expression on cardiomyocyte post-transplantation has been associated with increased cell cycle entry and proliferation during acute rejection episodes (111).

These paradoxical results can be related to differential expression of TNF receptors on distinct cell types which can thus lead to contradictory phenotypes (**Figure 3**). In addition, while some data is available on TNFR2 expression on immune regulatory cells in the context of solid organ transplantation, there remains many unstudied avenues to pursue. Finally, as a link between TNFR2 signaling and stem cell property induction in tubular epithelial cells has been demonstrated, research exploring therapeutic potentials for TNFR2 targeting in transplant rejection and modulation of ischemia reperfusion injury can be considered.

## Therapeutic Potential in Solid Organ Transplantation

The therapeutic potential of TNFR2 agonists and antagonists has been progressively more studied in the context of CD4+Treg expansion or depletion in both autoimmunity, GvHD, and cancer (121, 122). However, there remains a lack of studies in solid organ transplantation. In this section, we will discuss potential therapeutic avenues with regard to the emerging evidence available on TNFR2 pharmacological agents.

First, selective TNFR2 targeting has been increasingly study in the setting of GvHD (40, 123–126). Indeed, TNFR2 agonist treatment was shown to promote CD4+Treg expansion and improve resolution of rejection while TNFR2 antagonists abrogated these results. As CD4+Treg TNFR2 frequency has been associated with improved short term kidney transplantation outcomes, this could be an exciting avenue to follow. By doing so, both improved graft survival in solid organ transplantation as well as improved treatments against rejection could be possible.

Finally, as TNFR2 has been shown to induce stem cell properties of tubular epithelial cells and their subsequent regeneration (115). TNFR2 expression in mesenchymal stem cells has also been shown to maintain their regenerative functions and induce CD4+Tregs while TNFR2 inhibition has been shown to decrease expression of the mesenchymal stem cell characteristics (127). These novel findings could also allow for TNFR2 targeting in transplantation to induce epithelial regeneration. However, this will have to be done with care as TNFR2 expression varies greatly in different cell types and its effects can be quite dichotomous.

## Conclusion

The expression of TNFR2 on CD4+Tregs, CD8+Tregs, MDSCs, and Bregs has been associated with an immune regulatory phenotype. In solid organ transplantation, TNFR2+CD4+Tregs and TNFR2+MDSCs have been infrequently studied and have been associated with improved short-term outcomes after renal transplantation as well as increased CD4+Treg frequency and suppressive function. TNFR2 has also been observed on tubular epithelial cells as well as cardiomyocytes of renal and cardiac allografts respectively. In the context of tubular epithelial cells, recent evidence has shown a correlation between TNFR2 expression and stem cell phenotype induction while in the context of TNFR2 expression on cardiomyocytes, contradictory results have been observed.

This review highlights the positive impact that TNFα-TNFR2 signaling appears to have in solid transplantation. However, the scarcity of data requires further investigation in the role of TNFR2 in all types of solid organ transplantation as well as dedicated studies on the therapeutic use of TNFR2 agents during induction, maintenance therapy, or in the treatment of rejection.

## Author Contributions

AK, JT, and SP reviewed the literature. AK drafted the manuscript. All authors contributed to the editing of the manuscript. All authors contributed to the article and approved the submitted version.

## Conflict of Interest

The authors declare that the research was conducted in the absence of any commercial or financial relationships that could be construed as a potential conflict of interest.

## Publisher’s Note

All claims expressed in this article are solely those of the authors and do not necessarily represent those of their affiliated organizations, or those of the publisher, the editors and the reviewers. Any product that may be evaluated in this article, or claim that may be made by its manufacturer, is not guaranteed or endorsed by the publisher.
